# A Comparative Analysis of Lipid Digestion in Human Milk and Infant Formulas Based on Simulated In Vitro Infant Gastrointestinal Digestion

**DOI:** 10.3390/foods11020200

**Published:** 2022-01-12

**Authors:** Lu Liu, Shuang Lin, Shuaiyi Ma, Yue Sun, Xiaodong Li, Shuyan Liang

**Affiliations:** 1 Key Laboratory of Dairy Science, Ministry of Education, Northeast Agricultural University, No. 600 Changjiang St., Xiangfang District, Harbin 150030, China; liulu89824@163.com (L.L.); treeshuang@163.com (S.L.); ma_shuaiyi@163.com (S.M.); sy_neau@126.com (Y.S.); hansiwei2006@126.com (S.L.); 2 Food College, Northeast Agricultural University, No. 600 Changjiang St., Xiangfang District, Harbin 150030, China

**Keywords:** human milk, infant formula, particle size, fatty acid release, digestion

## Abstract

To investigate the lipid digestive behaviors of human and infant formulas and analyze the differences between them, we investigated the fat globule particle size distribution, lipolysis rate, and fatty acid release of infant formulas with different fat sources and human milk using an in vitro infant digestion model. The results suggested that the particle size in infant formula increased rapidly during gastric digestion and decreased significantly after intestinal digestion, whereas the particle size in human milk increased slowly during gastric digestion but increased rapidly during intestinal digestion (*p* < 0.05). Despite having a larger droplet size, human milk demonstrated a very high lipolysis rate due to the presence of MFGM. In terms of the distribution of fatty acids in digestion products, the proportion of saturated fatty acids (SFAs), monounsaturated fatty acids (MUFAs), and polyunsaturated fatty acids (PUFAs) in vegetable oil-based infant formulas was close to that of human milk. The amount of SFAs in milk fat-based infant formulas was significantly higher than that in human milk, and the content of MUFAs in all infant formulas was significantly lower than that in human milk (*p* < 0.05). After digestion, the most abundant fatty acid released by human milk was C18:2n6c, while the fatty acids released by infant formulas were SFAs, such as C14:0, C16:0, and C18:0.

## 1. Introduction

Human milk, as the source of all nutrients for infants in the first 6 months, meets all the energy requirements for their growth and development, and lipids are an important component. The fat content in human milk is about 3–5%, and it provides 45–55% of the energy requirements of an infant in the first 6 months [1]. Fat in human milk exists in the form of fat globules, with an average diameter of about 3–5 μm. Fat globules are composed of an inner core of triglycerides and milk fat globule membrane (MFGM) [2]. In addition, human milk fat contains a variety of PUFAs, phospholipids, fat-soluble vitamins, and other essential nutrients, which are beneficial to the growth and development of infants. These components promote the development of the nervous system and organs [3] and accelerate the synthesis of biofilms [4]. Several studies have also reported the role of milk fat in regulating gastrointestinal function and lipoprotein metabolism [5,6]. If breast-feeding is not possible or sufficient, it is necessary to substitute human milk with infant formula to provide the necessary nutrients.

At present, a variety of oils are mixed to simulate the composition of fatty acids in human milk, and vegetable oil and milk fat are the most common sources of fat in infant formulas [7]. In addition, a small amount of algal oil and fish oil are added to supplement PUFAs [5]. Although the addition of these fat components makes infant formulas similar to human milk in terms of certain important fatty acids and triglycerides, there is still a big gap in terms of the fat digestibility of infant formulas and human milk, as the structure of triglycerides and the size of fat globules also strongly affect fat absorption [8]. In vegetable oil-based infant formulas, fat globules are sub-micron size (0.4–0.5 μm) and wrapped in a thick layer of milk protein or other non-milk-derived surface-active emulsifiers (plant lecithin and monoglyceride). Some components cannot be completely digested by an infant [9]. For whole fat milk infant formulas, the fat globule structure will be destroyed by repeated homogenization and sterilization [10,11]. Moreover, vegetable oil and deep-sea fish oil are usually added to make up for the lack of unsaturated fatty acids, which makes the structure of fat globules more complicated. However, the addition of the MFGM components also affects the structure of the fat globules [12]. Therefore, the digestion and absorption of fat in different infant formulas are different. Recent studies have shown that the composition of fat in infant formulas plays an important role in the growth and development of infants, while the physical structure of fat globules also has special functions [13]. The particle size and interface composition of fat globules significantly affect their digestibility, which further affects the absorption and utilization of fat [14,15,16]. The results reported by Li et al. [16] have shown that the degree of lipolysis of small fat globules is higher, and there is a short lag period in the fat digestion profile in the emulsion of protein-coated droplets. Despite the abundance of research on human milk and infant formulas, such as on their chemical composition, their microstructure, and the free fatty acid release from vegetable and bovine milk fat-based infant formulas [12,17], little is known concerning the differences in lipid profiles and the distribution of fatty acids in human milk and infant formulas (especially in infant formulas with MFGM) and their effect on infant fat digestion.

Adults rely on pancreatic lipase to hydrolyze triglycerides. However, the development of the infant pancreas is still immature. As a result, the concentrations of pancreatic lipase and bile salts are relatively low, and their ability to hydrolyze triglycerides is very limited. In contrast, human gastric lipase (HGL), the sn-3 specific lipase that specifically hydrolyzes glycerol lipids to form free fatty acids and diglycerides, is highly secreted in infants [5,18]. It is resistant to pepsin and can be adsorbed on the interface of fat globules to hydrolyze fat [19]. In addition, it can improve the lipolysis capacity of pancreatic lipase, bile salt-stimulated lipase (BSSL), and pancrelipase-related protein 2 (PLRP2) [20,21]. However, there are few studies on the importance of gastric lipase; thus, the digestion of fat globules in the infant gastrointestinal tract is not fully understood. Lipase is an interfacial enzyme that can be adsorbed to the oil–water interface. It plays a catalytic role in the hydrolysis of fat. The smaller the fat globules are, the greater the total interface area will be and the more opportunities lipase will have to contact fat globules. This improves the efficiency of fat hydrolysis [22]. Therefore, the particle size of fat globules significantly affects the digestion of fat.

This study established a simulated in vitro infant digestion model based on the characteristics of the infant gastrointestinal tract and analyzed changes in the particle size, acylglycerol profile, and fatty acid release of human and commercial infant formulas. A comparative analysis was performed between the digestion characteristics of human and infant formulas from the perspective of fatty acid, which may offer a theoretical basis for improving infant formulas and help in designing new infant formulas to mimic human milk better.

## 2. Materials and Methods

### 2.1. Materials

Human milk was provided by 10 healthy Chinese women (18–30 years of age) who delivered at full term and had no history of drug use. Samples were collected at the Hospital, Song Yuan city, and were stored in cryogenic tubes (10 mL) at −80 °C until further chemical analysis.

Commercially available infant formulas, including two kinds of vegetable oil-based formulas (IF1 and IF2: IF1 without MFGM, IF2 with MFGM) and three kinds of milk/vegetable oil-based formulas (IF3, IF4, and IF5: IF3 with cream, IF4 with cream and soybean phospholipid, IF5 with no supplements), were selected for the study. The fat sources of the five infant formulas are listed in Table S1.

### 2.2. Chemicals

Thirty-seven component fatty acid methyl esters (FAMEs), thin-layer chromatography (TLC) standards (oleic acid, monoolein, diolein, triolein 1:1:1:1), and *Rhizopus oryzae* lipase were purchased from Sigma Aldrich (St. Louis, MO, USA). Porcine pancreatic lipase, pepsin, trypsin, and Ox gall salt were purchased from Beijing Solable Technology Co., Ltd. (Beijing, China). All other high-purity reagents were purchased from Sinopharm Chemical Reagent Co., Ltd. (Beijing, China).

### 2.3. Methods

#### 2.3.1. Simulated Infant Gastrointestinal Digestion In Vitro

The simulation of gastrointestinal digestion was carried out according to the INFOGEST 2.0 method published by Andre et al. [23] with the modifications reported by Laure et al. [24], in which the simulation was adjusted to be close to the infant gastrointestinal tract. The static digestion model was used in this study. IF1-5 was prepared with ultra-purified water as an emulsion with a fat content of 4.2% (*w*/*v*) to achieve the concentration of fat in human milk. *Rhizopus oryzae* lipase is acid-resistant and exhibits region-specific hydrolysis to HGL, which replaced gastric lipase in this study. *Rhizopus oryzae* lipase and porcine pepsin were added to the simulated gastric fluid (SGF) (72.2 mmol/L Na^+^, 7.8 mmol/L K^+^, 0.15 mmol/L Ca^2+^, 55 mmol/L Cl^−^) in concentrations of 19 U/mL and 268 U/mL, respectively. Bovine bile salts (3.1 mmol/L), porcine pancreatin (90 lipase U/mL), and porcine trypsin (16 U/mL) diluted in simulated intestinal fluid (SIF) (123.4 mmol/L Na^+^, 7.6 mmol/L K^+^, 0.6 mmol/L Ca^2+^, 47 mmol/L Cl^−^) were used.

In this study, we took 126 mL of a milk sample for digestion and 20 mL samples were collected every hour during digestion. Separate digestion tubes were prepared for different samples and different time points (except for 0 h) as follows: No. 1 (gastric digestion 1 h), No. 2 (gastric digestion 2 h), No. 3 (intestinal digestion 1 h), and No. 4 (intestinal digestion 2 h). Gastric digestion was conducted in a shaking incubator at 37 °C. The pH was adjusted to 5.3 with 0.1 M HCl. No. 1 and No. 2 digestion tubes were taken after 1 h and 2 h of digestion for microstructure study and fat analysis. Tubes No. 3 and No. 4 were analyzed after 2 h of gastric digestion. SIF at 37 °C was added for intestinal digestion, and the pH was adjusted to 6.6 with 0.1 M NaOH or 0.1 M HCl. After 1 h and 2 h of digestion, 4-(2-aminoethyl) benzene-1-sulfonyl fluoride hydrochloride (CAS No.: 30827-99-7) was added to the No. 3 and No. 4 digestion tubes to stop the reaction, before samples were taken for a microstructure study and fat analysis.

#### 2.3.2. Particle Size Analysis

The particle size distribution of the fat globules in all samples was determined using a S3500 laser particle size analyzer (Microtrac, Largo, FL, USA). The volume-weighted particle size distribution and mean diameter (D4,3) of fat globules in the samples were measured at room temperature when the shading degree reached 10%.

#### 2.3.3. Lipid Extraction and Analysis

Acylglycerol composition was analyzed using thin-layer chromatography (TLC) and a dual-wavelength flying spot thin-layer scanner (CS-9301PC, Shimadzu Corporation, Tokyo, Japan) [25]. Chloroform-methanol (2:1 *v*/*v*) was used to extract fat from gastrointestinal digestion tests; 5 mL of sample was mixed with 20 mL of chloroform-methanol (2:1 *v*/*v*) and 5 mL of 0.73% NaCl. Then, the mixture was ultrasonically extracted for 10 min and centrifuged at 5000 rpm/min for 10 min. Finally, the bottom organic phase was dried under a gentle nitrogen stream. Ten milligrams of the extracted fat was spotted onto the TLC plate and developed for 20 min in a solvent tank containing hexane/diethyl ether/acetic acid (60:17:0.2, *v*/*v*/*v*). TLC standards were used to identify each lipid class, and a dual-wavelength flying spot thin-layer scanner was employed to scan at 370 nm. The percentages of various compositions were calculated based on the peak areas.

Free fatty acids were separated and quantified according to the method of García et al. [26], with slight modification. The extracted fat was dissolved in chloroform (20 mg/mL) and then transferred to an aminopropyl silica gel column. The free fatty acids were eluted with acetic acid-ether solution (2%). Free fatty acids were methylated using KOH-methanol solution (0.5 mol/L). Finally, the FAMEs were analyzed by a gas chromatography-mass spectrometer (GC-MS) and the values are expressed in μmol/g total fatty acids. An Agilent 7000DGC/TQ GC-MS (Agilent, Palo Alto, USA), equipped with a DB-5 (60 m × 0.25 mm × 0.25 μm) column, was used to detect the free fatty acids. The gas chromatography conditions were as follows: inlet temperature, 250 °C; carrier gas, He; flow rate, 1.0 mL/min. The oven temperature was programmed as follows: it was initially held at 150 °C for 2 min, increased to 250 °C at a rate of 5 °C/min, and held at 250 °C for 10 min. The mass spectrometry conditions were as follows: MS ion source performed full scan at 225 °C; the analytes were ionized by electron bombardment (EI, electron energy 70 eV); scan mass range, 50–500 amu.

#### 2.3.4. Statistical Analysis

Qualitative Navigator, Version-B.08.00 (California, USA)was used to extract GC-MS data, and Excel was employed to pre-process the extracted data. One-way ANOVAs and Kruskal Wallis tests were performed on the obtained data using IBM SPSS Statistics, Version23.0 (San Diego, CA, USA). Partial least squares discriminant analysis (PLS-DA) was performed using Simca, Version14.1 (Umetrics, Sweden). Ordinary graphs were drawn using Origin, Version-2018 (Northampton, MA, USA) and heat maps were drawn using Morpheus tools (Wuhan, China). All tests were repeated six times.

## 3. Results and Discussion

### 3.1. Initial Fat Globule Particle Size of Infant Formulas and Human Milk

As shown in Table 1, IF1 (0.79 μm) and IF2 (0.46 μm) had the smallest fat globules, which may due to IF1 and IF2 being vegetable oil-based formulas for which a high homogenization pressure is used during production. Compared with IF1 and IF2, the fat globules in IF3 (1.29 μm), IF4 (1.61 μm), and IF5 (1.09 μm) that contain milk fat were relatively large in terms of particle size but significantly smaller than human milk fat globules (4.52 μm). The particle size of the human milk fat globules (average 4.52 μm) was significantly larger than of the infant formulas, and the particle size distributions were between 2.31 μm and 7.78 μm, which was consistent with the reports of Lopez et al. and Fondaco et al. [27,28]. Several studies have shown that the large particle size of human milk fat globules could help with lipid metabolism in infants [29,30].

**Figure 1:**
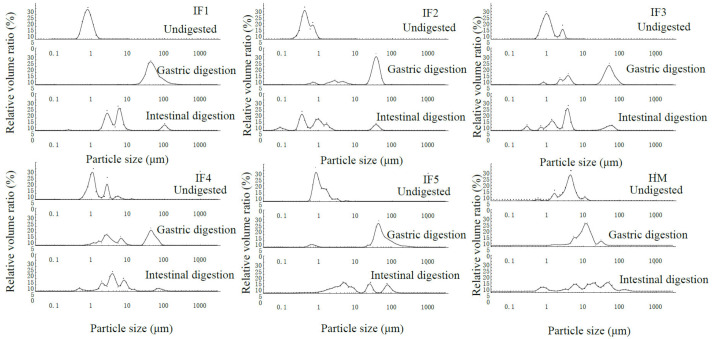
Particle size distribution of fat globules in infant formulas and human milk during digestion. IF1: vegetable oil-based formula; IF2: vegetable oil-based formula with MFGM; IF3: bovine milk/vegetable oil-based formula with cream; IF4 bovine milk/vegetable oil-based formula with cream and soybean phospholipid; IF5: whole fat milk power/vegetable oil-based formula.

Figure 1 reveals the differences in fat globule particle size distribution between infant formulas with different fat sources and human milk. The fat globules in IF1 showed a monomodal size distribution, while bimodal size distributions were detected in IF2. This may be due to the addition of MFGM components. In IF2, some fat globules were encapsulated by milk protein to form relatively small fat globules, while some fat globules were encapsulated by MFGM to form larger lipid droplets. The fat globules in IF3 (without phospholipid component) showed a bimodal size distribution, and those in IF4 (with soybean phospholipid) showed a trimodal size distribution. This may be because the fat globules formed by homogenization could not completely fuse with the vegetable oil added later; therefore, IF3 contained fat globules of two sizes. The trimodal distribution of fat globules in IF4 was due to the addition of soybean phospholipids, meaning that some of the fat globules were wrapped in plant phospholipids. The fat globule particle size distribution of IF5 (composed of whole fat milk powder and vegetable oil) was also uneven. This demonstrated that the particle size distribution of fat globules in the whole fat milk powder was also different from that of the milk supplemented with vegetable fat globules [10]. In addition, the number of fat globule particle size distribution peaks may depend on the number of emulsifiers used in the infant formula, which may also be the main reason for the variability in the fat globule particle size in infant formulas.

### 3.2. Fat Globule Particle Size Changes in Infant Formulas and Human Milk during In Vitro Digestion

As shown in Table 1, after gastric digestion the emulsified layer on the surface of fat globules was partially destroyed, and the lipid droplets aggregated. As a result, the particle size of fat globules in all samples showed an increasing trend. In addition, acidic pH conditions caused milk protein precipitation and aggregation, which led to an increase in the average particle size of fat globules [31]. Compared with human milk, the fat globule particle size in infant formulas increased significantly (*p* < 0.05) and the average particle size in infant formulas was more than 20 μm, while that of human milk was only 12.48 ± 1.29 μm. This indicates that the stability of infant formula fat globules after gastric digestion was significantly less than that of human milk, and the excessive accumulation of infant formula fat globules during gastric digestion may have certain adverse effects on fat digestion [32]. After intestinal digestion, the average particle size of all infant formula fat globules was significantly reduced compared with that of after gastric digestion (*p* < 0.05). This was due to the higher pH in the intestinal digestion, which dissociates the casein micelles that bind to the fat globule surface and, thereby, rapidly reduces the size of the lipid droplets encapsulated by milk protein. In addition, a large amount of fat in infant formulas was hydrolyzed into free fatty acids and released into the intestinal digestion. In contrast, the particle size of human milk after intestinal digestion (27.62 ± 2.27 μm) was larger than that during gastric digestion (12.48 ± 1.29 μm), and Luo et al. [33] also obtained a similar phenomenon. This was due to the unique fat globule membrane structure in human milk. The bile salts and pancreatic lipase in the intestinal digestion reduce the stability of the MFGM, leading to the polymerization of fat globules and a rapid increase in the particle size. With the hydrolysis of triglycerides, the particle size of fat globules gradually decreases.

In terms of the fat globule particle size distribution (Figure 1), IF1 still showed a monomodal size distribution after gastric digestion, while IF2, IF3, and IF4 showed a multimodal size distribution. This may be due to the presence of phospholipids, which led to different interface compositions of lipid droplets in infant formulas. Thus, the aggregation degree of fat globules was different during gastric digestion. IF5 consisted of whole fat milk powder. Although this also contains phospholipids, the fat globule particle size distribution was in the range of 10–100 μm and showed a monomodal size distribution. This may be due to the many homogenization and high-temperature treatments of the whole fat milk powder prior to the experiment. As a result, its fat globules were encapsulated by a large amount of thermally aggregated milk protein, meaning that phospholipids had little effect on the fat globule interface composition. During intestinal digestion, the particle size distribution of fat globules in all samples became multimodal and the majority of peaks appeared in the small-particle-size region.

In summary, the particle size of fat globules in infant formulas was much smaller than that in human milk, and its distribution was affected by the emulsifying components in the infant formula. In addition, the changes in the particle size of fat globules during digestion were not entirely dependent on its initial size and may be related to the interface composition of fat globules.

### 3.3. Fatty Acid Release in Infant Formulas and Human Milk during Digestion

#### 3.3.1. Analysis of Lipolysis Products

Table 2 shows the changes in acylglycerol compositions during in vitro simulated gastrointestinal lipolysis. For both human milk and infant formulas, the concentration of triglycerides (TGs) decreased and the concentrations of free fatty acids (FFAs), monoglycerides (MGs), and diglycerides (DGs) increased along with the lipolysis time. At the end of gastric digestion, there was no difference in the content of TGs, FFAs, DGs, and MGs in human milk and infant formula. Nevertheless, at the end of intestinal digestion a lower TGs concentration (2.60%) and higher FFAs (86.42%), MGs (4.21%), and DGs (6.77%) concentrations were found in human milk compared to in infant formula (except IF2). This indicates that there is a higher lipolysis rate in human milk fat compared to infant formula. IF2 had the highest lipolysis rate in infant formula, probably due to its small particle size. A recent study found that the fat globule particle size was significantly associated with the lipid digestibility and bioavailability in infants [16]. Moreover, the addition of MFGM makes the structure similar to that of human milk fat globules, which is more conducive to binding with lipase [28].

#### 3.3.2. Release Rate and Content of Fatty Acids

**Figure 2:**
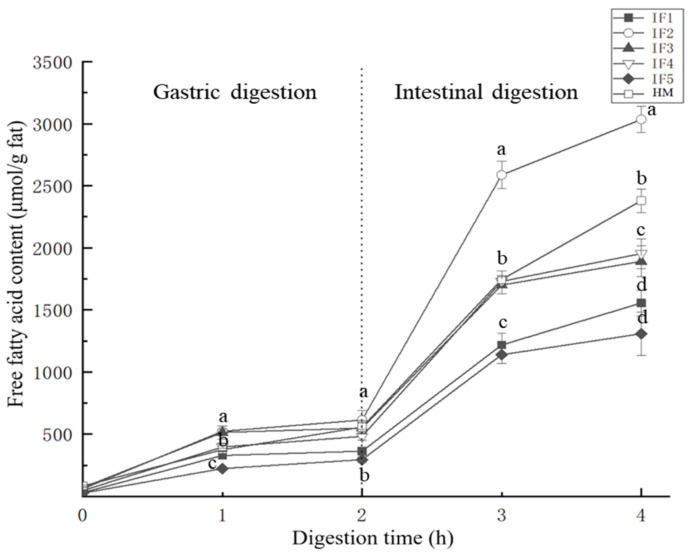
Free fatty acids released from infant formulas and human milk during digestion. IF1: vegetable oil-based formula; IF2: vegetable oil-based formula with MFGM; IF3: bovine milk/vegetable oil-based formula with cream; IF4 bovine milk/vegetable oil-based formula with cream and soybean phospholipid; IF5: whole fat milk power/vegetable oil-based formula. ^a–d^ Values with different superscripts differ significantly with respect to different samples.

The kinetics of the lipolysis of IFs and HM are shown in Figure 2. During the first hour of gastric digestion, the amount of fatty acids released and the release rate of each sample were significantly higher than in the second hour (*p* < 0.05). This may be due to the accumulation of fatty acids during digestion, which further inhibited the activity of gastric lipase [33] and reduced the substrate concentration. In addition, in the second hour of gastric digestion, compared with human milk, the release rate of fatty acids in infant formula was reduced more significantly. Since a large number of the fat droplets in infant formulas are covered by milk proteins with a strong hydrophobicity and electronegativity, gastric lipase was adsorbed relatively fast in the first hour of gastric digestion. However, the electronegativity of casein gradually decreased with hydrolysis [31], meaning that the fatty acid release rate of infant formulas differed greatly between the first and second hour of gastric digestion. The presence of MFGM in human milk reduced the contact between gastric lipase and triglycerides inside fat globules in the early stages of digestion, and as digestion progressed the MFGM was gradually destroyed [20] and more triglycerides were exposed to lipase. Although gastric lipase was inhibited by digestion products, human milk maintained a relatively high rate of fatty acid release.

During intestinal digestion, the release rate of fatty acids from all samples significantly increased (*p* < 0.05). This was due to the relatively high activity of pancreatic lipase in the intestinal digestion and the destruction of the surface of fat globules after gastric digestion. Additionally, a large amount of triglycerides were exposed, increasing the contact between pancreatic lipase and triglycerides. In the second hour of intestinal digestion, similar to gastric digestion, the release rate of fatty acids in infant formula was significantly reduced, while that in human milk remained relatively high.

In terms of the release of total fatty acids, IF2 released the highest concentration of free fatty acids (2973.97 ± 102.96 μmol/g), which exceeded that of human milk (2297.26 ± 86.97 μmol/g). This may be due to the smaller particle size of the fat globules, the larger contact area with lipase, and the higher lipolysis rate [22]. However, the release of fatty acids in other samples was not consistent with their initial particle size. The amount of fatty acids released from IF3 and IF4 was 1820.31 ± 119.87 μmol/g and 1906.79 ± 117.02 μmol/g, respectively, while the amounts released from IF1 and IF5 were 1524.94 ± 98.78 μmol/g and 1281.19 ± 166.85 μmol/g, respectively. The low concentration of fatty acids released from IF1 was likely related to its fat globules being covered by protein. Lipid droplets with this interface composition were prone to accumulate in large quantities in gastric digestion, which hindered complete hydrolysis in intestinal digestion [30]. The whole fat milk powder used in IF5 may be the cause of the low fatty acid release. According to the study of Lopez et al. [10] and Yao et al. [15], the use of multiple thermal processing steps leads to the formation of denatured protein hard shells on the outer layer of lipid droplets and affects the digestion of fat. The amount of fatty acids released during digestion is not only related to the particle size of fat globules but also to the interface composition of fat globules.

#### 3.3.3. The Content and Proportion of SFAs, MUFAs, and PUFAs

**Figure 3:**
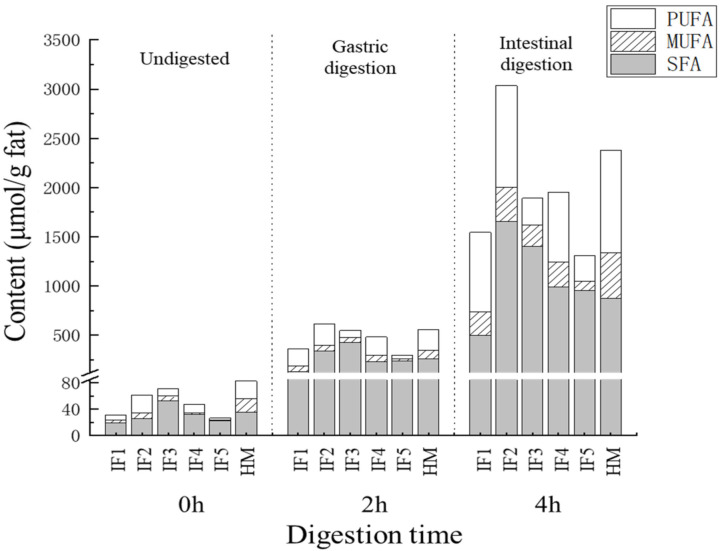
The content and proportion of SFA, MUFA, and PUFA in infant formulas and human milk during digestion. IF1: vegetable oil-based formula; IF2: vegetable oil-based formula with MFGM; IF3: bovine milk/vegetable oil-based formula with cream; IF4 bovine milk/vegetable oil-based formula with cream and soybean phospholipid; IF5: whole fat milk power/vegetable oil-based formula.

The content and proportion of SFAs, MUFAs, and PUFAs in infant formulas and human milk during digestion are shown in Figure 3. After gastric digestion, the most abundant type of fatty acids released by IF1 was PUFAs, accounting for 47.52% ± 2.66% of the total fatty acids, while the most abundant type in human milk and the other four infant formulas was SFAs. The proportions of SFAs in IF3 and IF5 reached 77.69% ± 4.53% and 72.01% ± 2.17%, respectively—significantly higher than that in human milk (47.19% ± 3.19%) (*p* < 0.05). This may because IF3 and IF5 were prepared from milk and whole fat milk powder, respectively. Raw milk contains a higher proportion of SFAs and a lower proportion of PUFAs than human milk [7]. Although IF4 was also prepared by adding cream and vegetable oil to milk, its proportion of SFAs after gastric digestion was relatively low (47.72% ± 2.76%), which may be due to the small amount of cream added in IF4.

After intestinal digestion, the proportion of SFAs decreased and the proportion of PUFAs increased in all samples. This phenomenon was most obvious in human milk, where the proportion of PUFAs (43.64% ± 4.75%) was greater than that of SFAs (36.68% ± 1.35%). This indicated that PUFAs were more inclined to release during intestinal digestion. The effect on the absorption of PUFAs was unclear. Compared with IF3 and IF5, which contain more milk fat, the release of PUFAs from IF1–2 and IF4, with more vegetable oil, was relatively higher. This was consistent with the low PUFA content and high SFA content of milk fat. In addition, compared with human milk the release of MUFAs in all infant formulas was significantly lower (*p* < 0.05), which may be related to the differences in triglyceride structure between infant formulas and human milk. Previously, many studies have reported that human milk has more MUFAs at sn-1,3 than vegetable oil and milk fat, and that these MUFAs are released more easily through hydrolysis [13].

Overall, the proportion of SFAs, MUFAs, and PUFAs in vegetable oil-based infant formulas was similar to that of human milk during digestion. Adding milk fat significantly increased the SFA content in digestion products (*p* < 0.05), which is worth noting when adding cream to infant formulas. The proportion of MUFAs in the final digestion products of the five infant formulas was significantly less than that of human milk (*p* < 0.05), which may affect the overall digestion and absorption of fat in infants.

#### 3.3.4. Composition of Free Fatty Acids

**Figure 4:**
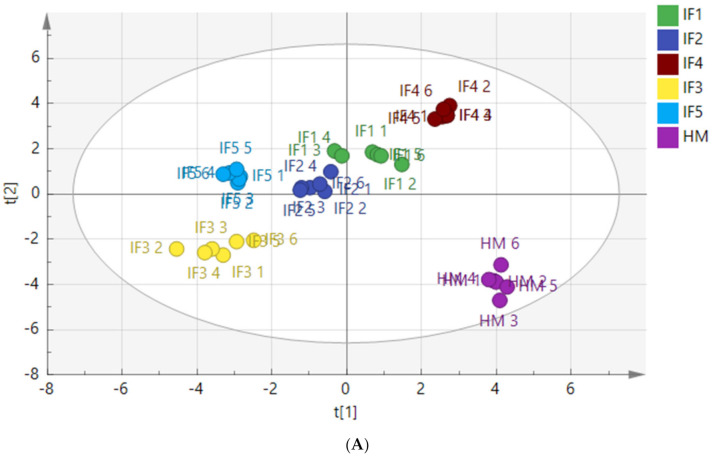

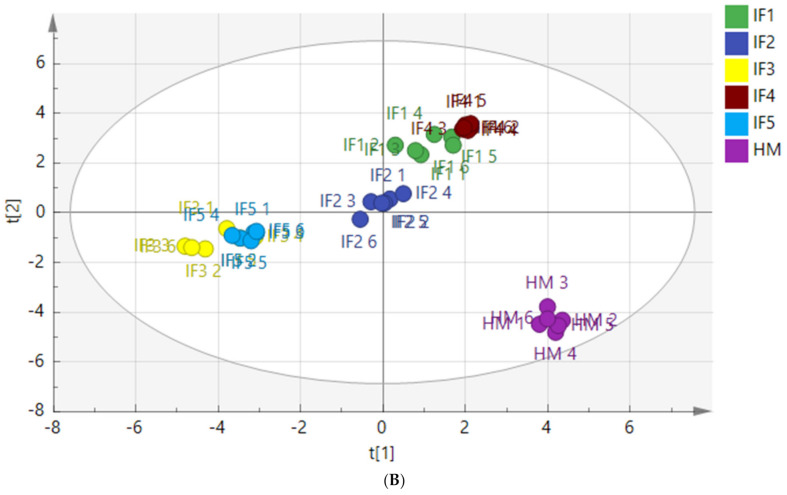
PLS-DA model score chart of fatty acids in infant formulas and human milk after gastric digestion (**A**) and intestinal digestion (**B**). IF1: vegetable oil-based formula; IF2: vegetable oil-based formula with MFGM; IF3: bovine milk/vegetable oil-based formula with cream; IF4 bovine milk/vegetable oil-based formula with cream and soybean phospholipid; IF5: whole fat milk power/vegetable oil-based formula.

To characterize the differences in fatty acid composition between human milk and infant formulas, PLS-DA and the Kruskal Wallis test were performed after the normalized pretreatment of free fatty acids produced by digestion. The PLS-DA score plots are shown in Figure 4.

Figure 4A shows a clear distinction between the fatty acids released from human milk and infant formulas after gastric digestion. The distribution of IF1 and IF2, with vegetable oil as a fat source, was similar, and the distribution of IF3 and IF5, with a high milk fat content, was similar. Figure 4B shows that the fatty acids released by human milk and infant formulas after intestinal digestion were significantly different. Although many infant formulas currently use a variety of oils to make their fatty acid composition as close to that of human milk as possible, the composition of fatty acids released after digestion remains significantly different from that of human milk. The vegetable oil-based IF1 and IF2 had similar distributions in the score chart, while the distributions of IF3 and IF5 with milk fat were close in the score chart.

The composition and content of fatty acid produced during digestion are shown in Table S2. It can be observed that, after gastric digestion, C18:2n6c was the most abundant fatty acid in human milk, accounting for 32.60% ± 2.17% of the total free fatty acids, followed by C16:0, C18:1, C12:0, C14:0, and C10:0, which was consistent with the high content of C18:2 in human milk [34]. Human milk also released a large amount of medium-chain saturated fatty acids (MC-SFAs) during gastric digestion. However, C14:0, C16:0, and C18:0 were the main fatty acids in infant formulas, especially in IF3 and IF5. The total proportions of these three fatty acids reached 68.45% ± 3.27% and 71.75% ± 4.76% in IF3 and IF5, respectively, and most of these were long-chain saturated fatty acids (LC-SFAs). Compared with LC-SFAs, MC-SFAs can directly enter the mitochondria for rapid oxidation and provide energy. Moreover, MC-SFAs effectively improve blood lipid metabolism and prevent obesity [35]. The beneficial effect of MC-SFAs on infant growth deserves further discussion.

After intestinal digestion, C18:2n6c and C18:1 were the most abundant fatty acids in human milk, and more than 65% of SFAs in human milk were MC-SFAs. It is also worth noting that the C16:0 produced by digestion only accounted for 7.64% of the total fatty acids, which was far lower than the total C16:0 content in human milk (21.08% ± 1.87%). The result was in accordance with previous research showing that the unsaturated fatty acids in human milk were mainly in the sn-1/3 positions of triglycerides, while SFAs such as C16:0 were mainly in the sn-2 position [36]. This triglyceride structure is of great significance for infant nutrient intake. It not only ensures the digestion and utilization of essential fatty acids but also reduces the production of calcium soaps and improves the utilization of calcium in infants. During intestinal digestion, C14:0, C16:0, and C18:0 were present in higher levels in infant formulas, and the proportion of these three fatty acids in IF3 and IF5 was more than 60%. This was consistent with the high content of these three fatty acids in milk. In addition, after intestinal digestion, the release of MUFAs in all infant formulas was lower than that in human milk, especially C18:1 (*p* < 0.05). Although many fats containing C18:1 have been added to infant formulas, the results of this study show that a large proportion of C18:1 in infant formulas was not released during gastrointestinal digestion. This may be due to the large amount of C18:1 in the sn-2 position of triglycerides in vegetable oil and milk fat, which are used as fat sources in infant formulas.

The VIP (variable importance in the projection) value of fatty acids represents its contribution rate, which can be used to distinguish different samples; the larger the VIP value is, the greater the contribution rate is. Fatty acids with a VIP > 1 and *p* < 0.05 were selected as the differential fatty acids during digestion and are shown in Table 3. There were 13 differential fatty acids in human milk and infant formulas with different fat sources after gastric digestion, including five SFAs, four MUFAs, and four PUFAs (C20:1, C23:0, C18:3, C16:1, C15:0, C22:1n9, C8:0, C11:0, C20:2, C22:2, C14:1, C21:0, and C20:3). There were twelve differential fatty acids (C20:1, C23:0, C18:3, C16:1, C15:0, C22:1n9, C8:0, C11:0, C20:2, C22:2, C14:1, C21:0, and C20:3) in all samples after intestinal digestion, including six SFAs, four MUFAs, and two PUFAs. Compared with the results of gastric digestion, after intestinal digestion only C6:0 and C17:1 showed any significant difference.

**Figure 5:**
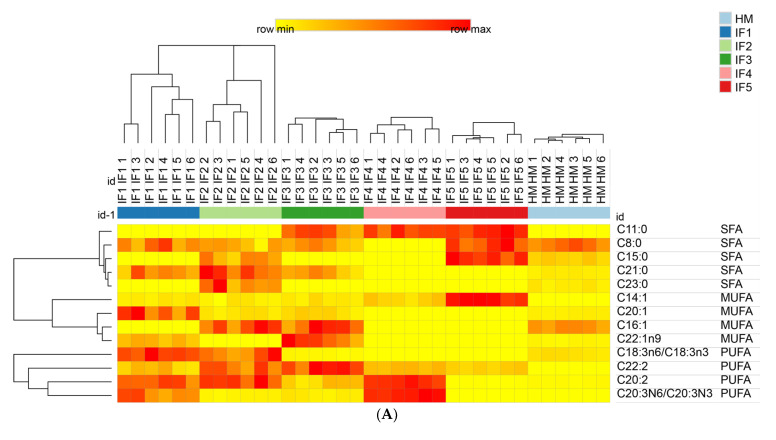

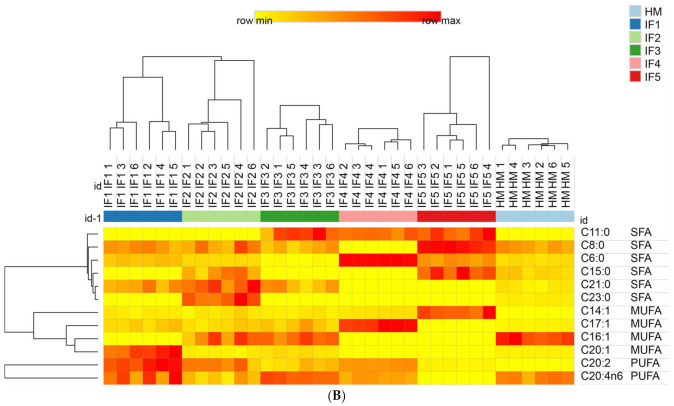
Distributions of differential fatty acids in infant formulas and human milk after gastric digestion (**A**) and intestinal digestion (**B**). IF1: vegetable oil-based formula; IF2: vegetable oil-based formula with MFGM; IF3: bovine milk/vegetable oil-based formula with cream; IF4 bovine milk/vegetable oil-based formula with cream and soybean phospholipid; IF5: whole fat milk power/vegetable oil-based formula. The grid in the heat map represents the relative content of a certain fatty acid. The color ranges from yellow to red, representing the content from low to high.

The fatty acids content in digestive products was further characterized by drawing heat maps and using hierarchical clustering; the results are shown in Figure 5. Figure 5A shows that the relative content of C22:2 in infant formulas was significantly higher than that in human milk. IF1 and IF2 released high levels of C18:3 and C20:2, while IF4, which contained a high amount of added vegetable oil, also released more C20:2. These findings indicate that infant formulas containing vegetable oil tend to release more C20:2 during gastric digestion, but this characteristic does not exist in human milk. IF3, IF4, and IF5 released high levels of C11:0, which was the common characteristic of milk/vegetable fat-based infant formulas. As shown in Figure 5B, after intestinal digestion the abundance of C16:1 was higher in human milk. IF1 had a significantly higher content of C20:1 and C20:2 (*p* < 0.05). This may be related to the higher content of rapeseed oil in IF1. IF2 had a significantly higher content of C21:0 and C23:0. IF3, IF4, and IF5 contained more C11:0 (*p* < 0.05), which is a significant feature of infant formulas with milk fat. In addition, the content of C6:0 and C17:1 in IF4 was significantly higher than that in the other samples (*p* < 0.05). IF5 had significantly more C8:0, C15:0, and C14:0 (*p* < 0.05), which may be related to the addition of coconut oil in IF5. After intestinal digestion, the fatty acid composition of infant formulas and human milk could no longer be clearly distinguished according to whether or not milk fat was added to infant formulas, and each infant formula demonstrated its own distinctive features in the fatty acid composition.

## 4. Conclusions

There were significant differences in the changes in the particle size of the fat globules in human milk and infant formulas during digestion; these are related to the different interface compositions of their fat globules. The gastrointestinal lipolysis rate depended not only on the particle size of fat globules but also on the interface composition. In addition to human milk, IF2 with MFGM also had a high lipolysis rate, indicating that the surface composition of fat globules affects the hydrolysis of fat. Regarding the content of SFAs, MUFAs, and PUFAs after digestion, the vegetable oil-based infant formulas (IF1, IF2) were close to human milk, while the infant formulas with milk fat had the highest SFA content. In the final digestion products, the proportion of MUFAs in all infant formulas was significantly lower than in human milk, which may affect the overall digestion and absorption of fat in infants. This comprehensive analysis of human milk and infant formula in lipid digestion can be used to guide the design of infant milk powders. The particle size and surface composition of fat globules should be considered when designing infant formulas.

## Figures and Tables

**Table 1:** Average particle size (μm) of fat globules in infant formulas and human milk.

Digestion Stage	IF1	IF2	IF3	IF4	IF5	Human Milk
Undigested	0.79 ± 0.05 ^c^	0.46 ± 0.03 ^c^	1.29 ± 0.12 ^c^	1.61 ± 0.09 ^c^	1.09 ± 0.11 ^c^	4.52 ± 0.37 ^c^
After gastric digestion	56.31 ± 2.66 ^a^	28.55 ± 2.37 ^a^	38.33 ± 1.29 ^a^	24.09 ± 1.33 ^a^	60.18 ± 3.85 ^a^	12.48 ± 1.29 ^b^
After intestinal digestion	15.34 ± 0.72 ^b^	5.16 ± 0.96 ^b^	11.06 ± 0.63 ^b^	9.55 ± 0.82 ^b^	23.42 ± 1.38 ^b^	27.62 ± 2.27 ^a^

^a–c^ Values with different superscripts differ significantly with respect to different samples (*p* < 0.05). IF1: vegetable oil-based formula; IF2: vegetable oil-based formula with MFGM; IF3: bovine milk/vegetable oil-based formula with cream; IF4 bovine milk/vegetable oil-based formula with cream and soybean phospholipid; IF5: whole fat milk power/vegetable oil-based formula.

**Table 2:** Changes in the acylglycerol profile during the in vitro gastrointestinal lipolysis of human milk and infant formulas.

Simple	Fat	Undigested	Gastric Digestion		Intestinal Digestion	
		0 h	1 h	2 h	3 h	4 h
HM	TGs	97.16 ± 4.83 ^a^	62.10 ± 3.45 ^b^	55.20 ± 2.99 ^b^	18.44 ± 2.25 ^c^	2.62 ± 1.68 ^d^
	DGs	2.84 ± 0.42 ^c^	12.20 ± 1.65 ^a^	9.57 ± 1.67 ^b^	8.82 ± 1.86 ^b^	7.12 ± 1.44 ^b^
	MGs	0	6.42 ± 0.97 ^b^	6.82 ± 1.28 ^b^	8.78 ± 0.86 ^a^	4.22 ± 0.87 ^c^
	FFAs	0	19.28 ± 2.23 ^d^	28.41 ± 3.24 ^c^	63.96 ± 3.98 ^b^	86.04 ± 4.16 ^a^
IF1	TGs	99.38 ± 4.43 ^a^	63.45 ± 3.52 ^b^	58.18 ± 2.29 ^b^	22.56 ± 2.12 ^c^	5.67 ± 1.55 ^d^
	DGs	0.62 ± 0.22 ^c^	11.64 ± 1.45 ^a^	10.23 ± 1.78 ^a^	10.62 ± 2.76 ^a^	8.42 ± 1.33 ^b^
	MGs	0	7.03 ± 1.85 ^b^	6.37 ± 1.23 ^b^	7.98 ± 1.67 ^a^	7.03 ± 0.87 ^b^
	FFAs	0	17.88 ± 2.11 ^d^	25.22 ± 2.24 ^c^	58.84 ± 2.88 ^b^	78.88 ± 3.01 ^a^
IF2	TGs	99.23 ± 5.13 ^a^	61.08 ± 3.02 ^b^	54.78 ± 3.69 ^b^	16.26 ± 2.22 ^c^	2.24 ± 1.35 ^d^
	DGs	0.77 ± 0.12 ^d^	11.12 ± 2.15 ^a^	8.92 ± 1.88 ^b^	7.84 ± 1.82 ^b^	6.36 ± 1.23 ^c^
	MGs	0	7.78 ± 1.23 ^a^	7.37 ± 1.57 ^a^	6.98 ± 1.67 ^a^	2.73 ± 0.64 ^b^
	FFAs	0	20.02 ± 1.11 ^c^	28.93 ± 2.24 ^c^	68.92 ± 3.98 ^b^	88.67 ± 5.31 ^a^
IF3	TGs	99.11 ± 5.13 ^a^	61.42 ± 3.02 ^b^	58.46 ± 2.66 ^b^	19.22 ± 2.24 ^c^	3.45 ± 1.35 ^d^
	DGs	0.89 ± 0.42 ^c^	11.78 ± 3.15 ^a^	10.43 ± 2.88 ^b^	10.06 ± 2.43 ^b^	9.53 ± 2.34 ^b^
	MGs	0	6.92 ± 1.54 ^a^	6.23 ± 1.43 ^a^	7.00 ± 2.77 ^a^	4.61 ± 1.64 ^b^
	FFAs	0	19.88 ± 1.89 ^d^	24.88 ± 2.64 ^c^	63.72 ± 3.98 ^b^	82.41 ± 4.66 ^a^
IF4	TGs	99.02 ± 5.83 ^a^	61.80 ± 3.45 ^b^	57.37 ± 2.99 ^b^	18.72 ± 2.25 ^c^	3.88 ± 0.68 ^d^
	DGs	0.98 ± 0.34 ^d^	11.84 ± 2.65 ^a^	9.82 ± 1.67 ^b^	10.06 ± 2.86 ^b^	8.74 ± 1.44 ^c^
	MGs	0	6.81 ± 1.97 ^a^	6.78 ± 2.28 ^a^	7.24 ± 2.86 ^a^	4.21 ± 0.87 ^b^
	FFAs	0	19.55 ± 2.23 ^d^	26.03 ± 3.24 ^c^	63.98 ± 3.79 ^b^	83.17 ± 5.66 ^a^
IF5	TGs	98.74 ± 4.32 ^a^	65.22 ± 3.16 ^b^	59.24 ± 2.69 ^b^	23.02 ± 2.12 ^c^	7.05 ± 1.66 ^d^
	DGs	1.26 ± 0.12 ^c^	12.10 ± 1.15 ^a^	10.28 ± 1.78 ^b^	12.62 ± 2.76 ^a^	12.50 ± 1.43 ^a^
	MGs	0	6.26 ± 0.85 ^b^	6.02 ± 1.23 ^b^	7.34 ± 0.67 ^a^	6.22 ± 0.58 ^b^
	FFAs	0	16.42 ± 1.11 ^d^	24.46 ± 1.24 ^c^	57.02 ± 1.97 ^b^	74.23 ± 4.01 ^a^

^a–d^ Values with different superscripts differ significantly with respect to different digestive stage (*p* < 0.05). IF1: vegetable oil-based formula; IF2: vegetable oil-based formula with MFGM; IF3: bovine milk/vegetable oil-based formula with cream; IF4 bovine milk/vegetable oil-based formula with cream and soybean phospholipid; IF5: whole fat milk power/vegetable oil-based formula.

**Table 3:** Differential fatty acids of infant formulas and human milk during digestion.

Digestion	Variable Name	VIP Value	*p* Value
Gastric digestion	C20:1	1.32404	0.0000054
	C23:0	1.27437	0.0000018
	C18:3n6/C18:3n3	1.12550	0.0000028
	C16:1	1.12003	0.0000078
	C15:0	1.09888	0.0000025
	C22:1n9	1.08643	0.0000023
	C8:0	1.07105	0.0000864
	C11:0	1.05986	0.0000071
	C20:2	1.03670	0.0000073
	C22:2	1.02923	0.0000106
	C14:1	1.02481	0.0000312
	C21:0	1.01863	0.0000049
	C20:3n6/C20:3n3	1.01797	0.0000028
Intestinal digestion	C23:0	1.49	0.0000018
	C20:1	1.36	0.0000032
	C21:0	1.23	0.0000051
	C6:0	1.14	0.0000113
	C17:1	1.12	0.0000095
	C11:0	1.06	0.0000080
	C20:4n6	1.05	0.0000365
	C16:1	1.04	0.0000071
	C8:0	1.03	0.0000813
	C20:2	1.03	0.0000039
	C15:0	1.02	0.0000031
	C14:1	1.00	0.0000376

## Supplementary Materials

The following supporting information can be downloaded at: https://www.mdpi.com/article/10.3390/foods11020200/s1. Table S1: The composition of infant formulas; Table S2: The content of differential fatty acids in IF1-IF5 before and after digestion.
