# Supplementary material for: A Comparative Analysis of Lipid Digestion in Human Milk and Infant Formulas Based on Simulated In Vitro Infant Gastrointestinal Digestion
Source: Foods. 2022 Jan 12;11(2):200. doi: 10.3390/foods11020200 (PMC8774497; doi:10.3390/foods11020200)
Supplement: Supplementary file 1 [file foods-11-00200-s001.zip › foods-1448216-supplementary.pdf]

Table S1. The composition of infant formulas.

Sample	Main milk base	Main source of fat	Source of Protein	Carbohydrate	Vitamins	Minerals
IF1	Skimmed milk powder	Vegetable oil (Palm oil, rapeseed oil, coconut oil and sunflower oil), OPO, DHA, ARA	Whey protein powder	Galactool-igosaccharide, Fructool-igosaccharide	Vitamins A, C, D, E, K1, B1, B2, B3, B5, B6, B7	Na^+, k^+, Cu^{2+}, Mg^{2+}, Fe^{2+}, Zn^{2+}, Mn^{2+}, Ca^{2+}, P^{3+}, I^-, Cl^-, Se^{4+}
IF2	Skimmed milk powder	Vegetable oil (Palm oil, rapeseed oil, coconut oil and sunflower oil), OPO, DHA, ARA, MFGM	Desalted whey powder, Casein phosphopeptide,	Galactool-igosaccharide, Polydextrose	Vitamins A, C, D, E, K1, B1, B2, B3, B5, B6, B7	Na^+, k^+, Cu^{2+}, Mg^{2+}, Fe^{2+}, Zn^{2+}, Mn^{2+}, Ca^{2+}, P^{3+}, I^-, Cl^-, Se^{4+}
IF3	Raw milk	Vegetable oil (Sunflower seed oil, rapeseed oil and coconut oil), Cream, OPO	Desalted whey powder, Casein phosphopeptide	Lactose, Fructool-igosaccharide	Vitamins A, C, D, E, K1, B1, B2, B3, B5, B6, B7	Na^+, k^+, Cu^{2+}, Mg^{2+}, Fe^{2+}, Zn^{2+}, Mn^{2+}, Ca^{2+}, P^{3+}, I^-, Cl^-, Se^{4+}
IF4	Raw milk	Vegetable oil (Sunflower seed oil, rapeseed oil, walnut oil and coconut oil), Cream, OPO, DHA, ARA, Phospholipid	Desalted whey powder, Whey protein powder	Lactose, Fructool-igosaccharide	Vitamins A, C, D, E, K1, B1, B2, B3, B5, B6, B7	Na^+, k^+, Cu^{2+}, Mg^{2+}, Fe^{2+}, Zn^{2+}, Mn^{2+}, Ca^{2+}, P^{3+}, I^-, Cl^-, Se^{4+}
IF5	Whole fat milk powder	Vegetable oil (Soybean oil, corn oil, coconut oil, sunflower oil and rapeseed oil), DHA, ARA	Desalted whey powder, Casein phosphopeptide	Galactool-igosaccharide, Fructool-Igosaccharide, Lactose	Vitamins A, C, D, E, K1, B1, B2, B3, B5, B6, B7	Na^+, k^+, Cu^{2+}, Mg^{2+}, Fe^{2+}, Zn^{2+}, Mn^{2+}, Ca^{2+}, P^{3+}, I^-, Cl^-, Se^{4+}

Table S2. The content of differential fatty acids in IF1~IF5 before and after digestion.

Types of fatty acids	Keep time（min）	content（μmol/g fat）		
		0 h (before digestion)	2 h (gastric digestion)	4 h (intestinal digestion)
		**IF1**		
C6:0	6.749	0.19±0.07^c	3.01±1.2^b	5.48±0.77^a
C8:0	10.304	0.45±0.27^c	2.9±1.42^b	10.95±2.73^a
C10:0	13.293	0.7±0.66^c	9.98±6.1^b	39.56±9.78^a
C11:0	14.632	——	——	——
C12:0	15.895	0.85±0.5^c	6.41±4.8^b	33.9±11.2^a

C13:0	17.089	——	——	——
C14:1	18.100	0.69±0.27[c]	4.12±1.53[b]	18.24±5.49[a]
C14:0	18.221	3.27±0.73[c]	28.34±8.91[b]	120.5±14.14[a]
C15:1	19.188	——	——	——
C15:0	19.299	——	——	——
C16:1	20.136	——	——	——
C16:0	20.339	6.99±2.36[c]	48.96±8.38[b]	166.23±49.27[a]
C17:1	21.273	0.65±0.2[c]	3.39±0.82[b]	16.31±4.22[a]
C17:0	21.514	0.51±0.15[c]	3.06±1.33[b]	12.51±5.37[a]
C18:3	22.288	3.74±1.36[c]	61.93±18.34[b]	230.96±48.24[a]
C18 : 2n6t	22.511	1.02±0.32[c]	25.41±8.93[b]	125.85±18.95[a]
C18 : 2n6c	22.607	0.69±0.35[c]	11.94±3.71[b]	48.33±20.28[a]
C18 : 1	22.680	2.40±1.12[c]	37.31±14.33[b]	144.69±33.27[a]
C18:0	22.975	5.33±1.38[c]	27.14±3.45[b]	94.58±14.51[a]
C20 : 4n6	25.664	——	0.66±0.25[b]	8.83±3.19[a]
C20 : 5n3	25.819	——	——	——
C20:3n6/C20:3n3	26.07	0.26±0.05[c]	37.18±6.5[b]	172.03±41.85[a]
C20:2	26.534	1.00±0.33[c]	29.51±7.46[b]	200.46±36.68[a]
C20:1	26.665	0.85±0.40[c]	9.6±2.98[b]	51.04±11.33[a]
C20:0	27.352	0.47±0.09[c]	2.52±0.56[b]	7.98±3.95[a]
C21:0	30.708	0.28±0.12	1.5±0.55	4.85±1.43
C22 : 6n3	31.666	0.2±0.07[c]	1.54±0.94[b]	6.07±2.58[a]
C22:2	33.721	0.35±0.10[c]	4.31±1.97[b]	17.79±4.11[a]
C22:1n9	33.914	——	2.24±0.50[b]	8.69±2.77[a]
C22:0	35.22	——	——	——
C23:0	41.217	——	——	——
C24:1	44.554	——	——	——
C24:0	45.633	——	——	——
Total free fatty acids		30.99±3.78[c]	365.97±30.12[b]	1545.93±99.56[a]
		**IF2**		
C6:0	6.749	0.20±0.02[c]	1.10±0.12[b]	8.35±3.23[a]
C8:0	10.304	0.50±0.18[c]	2.94±1.48[b]	22.50±7.05[a]
C10:0	13.293	1.76±0.52[c]	16.59±6.37[b]	103.32±37.2[a]
C11:0	14.632	——	——	——
C12:0	15.895	1.45±0.25[c]	10.29±1.73[b]	85.53±27.42[a]
C13:0	17.089	——	——	——
C14:1	18.100	0.82±0.34[c]	5.4±4.04[b]	47.14±16.28[a]
C14:0	18.221	4.98±0.93[c]	39.88±11.26[b]	289.4±87.74[a]
C15:1	19.188	——	——	——
C15:0	19.299	0.58±0.25[c]	3.06±0.98[b]	12.12±4.94[a]
C16:1	20.136	1.15±0.33[c]	8.09±2.73[b]	38.06±15.69[a]
C16:0	20.339	1.15±0.33[c]	137.61±38.77[b]	610.05±301.13[a]
C17:1	21.273	0.71±0.14[c]	5.81±2.49[b]	25.76±5.65[a]
C17:0	21.514	0.50±0.10[c]	3.67±1.26[b]	15.00±5.64[a]

C18:3	22.288	10.09±2.15[c]	77.61±30.25[b]	298.26±69.07[a]
C18：2n6t	22.511	5.99±2.01[c]	38.73±15[b]	219.49±69.39a
C18：2n6c	22.607	2.23±0.52[c]	12.35±2.43[b]	132.02±39.5[a]
C18：1	22.680	5.48±1.91[c]	37.90±11.40[b]	223.71±59.03[a]
C18:0	22.975	12.94±3.77[c]	114.35±39.01[b]	469.71±160.03[a]
C20：4n6	25.664	0.02±0.00[c]	0.60±0.19[b]	6.15±2.47[a]
C20：5n3	25.819	——	——	——
C20:3n6/C20:3n3	26.07	1.02±0.49[c]	6.27±1.69[b]	46.51±14.48[a]
C20:2	26.534	4.90±0.94[c]	60.25±16.3[b]	231.27±72.04[a]
C20:1	26.665	0.49±0.19[c]	2.61±0.81[b]	9.05±3.5[a]
C20:0	27.352	0.51±0.16[c]	2.76±1.13[b]	11.21±4.98[a]
C21:0	30.708	0.79±0.23[c]	4.10±1.04[b]	17.05±5.46[a]
C22：6n3	31.666	0.35±0.16[c]	2.02±0.91[b]	9.90±4.81[a]
C22:2	33.721	2.36±0.46[c]	18.71±5.71[b]	89.15±30.45[a]
C22:1n9	33.914	——	——	——
C22:0	35.22	——	——	——
C23:0	41.217	0.16±0.03[c]	1.47±0.75[b]	13.69±1.61[a]
C24:1	44.554	——	——	——
C24:0	45.633	——	——	——
Total free fatty acids		61.23±4.65[c]	614.29±74.58[b]	3035.20±457.61[a]
		**IF3**		
C6:0	6.749	0.20±0.08[c]	1.06±0.50[b]	3.24±1.68[a]
C8:0	10.304	0.55±0.18[c]	2.98±0.93[b]	9.25±2.75[a]
C10:0	13.293	2.51±0.85[c]	16.7±4.71[b]	57.2±24.71[a]
C11:0	14.632	1.22±0.33[c]	6.63±2.03[b]	22.64±8.2[a]
C12:0	15.895	1.77±0.87[c]	10.85±3.39[b]	34.97±13.02[a]
C13:0	17.089	——	——	——
C14:1	18.100	1.20±0.36[c]	6.49±2.02[b]	23.68±5.91[a]
C14:0	18.221	4.31±1.85[c]	27.28±12.51[b]	89.31±56.36[a]
C15:1	19.188	——	——	——
C15:0	19.299	——	——	——
C16:1	20.136	1.16±0.43[c]	7.40±2.64[b]	27.19±4.28[a]
C16:0	20.339	22.21±4.3[c]	173.51±51.25[b]	588.76±145.43[a]
C17:1	21.273	1.00±0.2[c]	6.74±2.26[b]	24.34±8.77[a]
C17:0	21.514	0.88±0.34[c]	5.73±0.78[b]	18.69±7.88[a]
C18:3	22.288	0.6±0.2[c]	3.95±1.03[b]	13.96±5.61[a]
C18：2n6t	22.511	1.83±0.69[c]	10.4±3.17[b]	37.03±14.87[a]
C18：2n6c	22.607	0.88±0.32[c]	4.85±1.82[b]	19.6±2.08[a]
C18：1	22.680	1.44±0.56[c]	7.61±1.99[b]	24.32±7.12[a]
C18:0	22.975	17.86±5.27[c]	174.98±48.33[b]	554.16±148.09[a]
C20：4n6	25.664	0.44±0.16[c]	2.80±0.95[b]	10.97±2.07[a]
C20：5n3	25.819	——	——	——
C20:3n6/C20:3n3	26.07	1.35±0.35[c]	8.84±2.66[b]	33.75±10.53[a]
C20:2	26.534	2.32±0.72[c]	15.67±7.19[b]	60.4±18.61[a]

C20:1	26.665	0.62±0.21[c]	3.21±1.14[b]	10.66±2.46[a]
C20:0	27.352	0.69±0.19[c]	4.62±0.95[b]	14.54±4.92[a]
C21:0	30.708	0.30±0.07[c]	1.67±0.68[b]	5.33±2.55[a]
C22：6n3	31.666	0.48±0.17[c]	3.47±0.9[b]	12.25±2.47[a]
C22:2	33.721	3.46±1.79[c]	22.76±5.63[b]	84.45±24.78[a]
C22:1n9	33.914	0.90±0.23[c]	8.17±2.74[b]	70.97±23.94[a]
C22:0	35.22	——	——	——
C23:0	41.217	——	——	——
C24:1	44.554	1.09±0.45[c]	10.13±4.39[b]	37.17±12.26[a]
C24:0	45.633	0.02±0.01[c]	0.47±0.2[b]	2.78±1.13[a]
Total free fatty acids		71.43±4.35[c]	549.13±47.4[b]	1891.74±234.22[a]
		**IF4**		
C6:0	6.749	0.42±0.06[c]	6.12±1.37[b]	18.62±1.53[a]
C8:0	10.304	0.35±0.17[c]	1.66±0.05[b]	5.58±0.03[a]
C10:0	13.293	1.81±0.07[c]	20.81±1.9[b]	90.35±6.14[a]
C11:0	14.632	1.15±0.09[c]	6.96±1.18[b]	24.47±2.75[a]
C12:0	15.895	1.36±0.11[c]	12.29±2.32[b]	43.19±9.14[a]
C13:0	17.089	——	——	——
C14:1	18.100	1.04±0.17[c]	10.49±3.13[b]	43.73±5.77[a]
C14:0	18.221	8.74±0.3[c]	72.05±4.31[b]	310.27±27.63[a]
C15:1	19.188	——	——	——
C15:0	19.299	——	——	——
C16:1	20.136	——	——	——
C16:0	20.339	10.52±0.86[c]	67.53±2.32[b]	313.66±10.58[a]
C17:1	21.273	1.36±0.26[c]	12.49±0.34[b]	40.58±0.40[a]
C17:0	21.514	——	4.56±0.14[b]	12.22±0.15[a]
C18:3	22.288	4.22±0.49[c]	83.05±0.65[b]	294.46±2.67[a]
C18：2n6t	22.511	3.48±0.21[c]	23.78±2.92[b]	120.81±5.09[a]
C18：2n6c	22.607	1.77±0.17[c]	11.51±2.52[b]	52.92±4.27[a]
C18：1	22.680	——	41.47±2.77[b]	158.56±10.31[a]
C18:0	22.975	6.74±0.35[c]	34.35±2.71[b]	159.05±7.84[a]
C20：4n6	25.664	——	1.79±0.58[b]	8.1±0.98[a]
C20：5n3	25.819	——	——	——
C20:3n6/C20:3n3	26.07	——	8.29±0.19[b]	32.79±0.19[a]
C20:2	26.534	2.07±0.13[c]	47.83±3.54[b]	166.28±7.66[a]
C20:1	26.665	——	2.19±0.02[b]	12.28±0.23[a]
C20:0	27.352	0.47±0.05[c]	2.42±0.25[b]	7.36±1.14[a]
C21:0	30.708	——	1.59±0.02[b]	6.78±0.06[a]
C22：6n3	31.666	0.53±0.02[c]	2.97±0.32[b]	9.62±2.11[a]
C22:2	33.721	1.66±0.12[c]	6.51±0.52[b]	22.8±3.09[a]
C22:1n9	33.914	——	——	——
C22:0	35.22	——	——	——
C23:0	41.217	——	——	——
C24:1	44.554	——	——	——

C24:0	45.633	——	——	——
Total free fatty acids		47.69±2.74[c]	482.71±33.49[b]	1954.48±109.76[a]
**IF5**				
C6:0	6.749	0.12±0.04[c]	1.16±0.25[b]	6.45±1.45[a]
C8:0	10.304	0.57±0.24[c]	3.22±0.83[b]	19.23±2.12[a]
C10:0	13.293	0.84±0.23[c]	5.2±0.87[b]	27.71±6.13[a]
C11:0	14.632	0.95±0.20[c]	4.93±0.66[b]	15.32±3.5[a]
C12:0	15.895	1.03±0.32[c]	5.26±1.12[b]	53.57±6.36[a]
C13:0	17.089	——	——	——
C14:1	18.100	0.89±0.4[c]	5.41±1.42[b]	31.01±3.70[a]
C14:0	18.221	5.39±0.78[c]	32.01±4.14[b]	128.65±31.9[a]
C15:1	19.188	——	——	——
C15:0	19.299	0.57±0.23[c]	3.04±0.51[b]	9.26±2.09[a]
C16:1	20.136	——	——	——
C16:0	20.339	1.38±0.46[c]	103.46±3.43[b]	382.68±42.95[a]
C17:1	21.273	——	5.01±0.07[b]	26.71±0.42[a]
C17:0	21.514	0.36±0.08[c]	1.88±0.6[b]	6.18±1.19[a]
C18:3	22.288	——	4.07±0.04[b]	17.29±0.12[a]
C18：2n6t	22.511	1.75±0.53[c]	15.39±1.23[b]	105.48±19.48[a]
C18：2n6c	22.607	0.68±0.14[c]	4.36±1.33[b]	70.48±8.17[a]
C18：1	22.680	0.37±0.10[c]	6.96±0.64[b]	26.95±4.55[a]
C18:0	22.975	10.53±2.46[c]	76.35±4.32[b]	294.48±32.4[a]
C20：4n6	25.664	——	1.02±0.03[b]	6.20±0.09[a]
C20：5n3	25.819	——	——	——
C20:3n6/C20:3n3	26.07	——	6.29±0.04[b]	23.52±0.72[a]
C20:2	26.534	0.04±0.01[c]	0.74±0.15[b]	12.02±2.41[a]
C20:1	26.665	——	1.74±0.04[b]	11.31±0.22[a]
C20:0	27.352	0.49±0.11[c]	2.55±0.75[b]	8.61±1.07[a]
C21:0	30.708	——	1.21±0.02[b]	3.21±0.03[a]
C22：6n3	31.666	0.21±0.07[c]	1.15±0.24[b]	4.43±0.78[a]
C22:2	33.721	0.47±0.06[c]	2.83±0.39[b]	17.08±1.46[a]
C22:1n9	33.914	——	——	——
C22:0	35.22	——	——	——
C23:0	41.217	——	——	——
C24:1	44.554	——	——	——
C24:0	45.633	——	——	——
Total free fatty acids		26.64±6.46[c]	295.24±22.99[b]	1307.83±173.31[a]
**Human milk**				
C6:0	6.749	——	1.44±0.57[b]	6.40±1.68[a]
C8:0	10.304	0.66±0.15[c]	4.70±0.87[b]	15.2±2.96[a]
C10:0	13.293	0.79±0.20[c]	19.63±2.85[b]	82.29±10.22[a]
C11:0	14.632	——	——	0.21±0.06[a]
C12:0	15.895	5.24±0.85[c]	76.42±5.22[b]	275.18±92.97[a]
C13:0	17.089	——	0.26±0.06[b]	1.07±0.18[a]

C14:1	18.100	——	0.11±0.01^{b}	0.63±0.17^{a}
C14:0	18.221	2.71±0.42^{c}	55.29±8.48^{b}	204.01±35.28^{a}
C15:1	19.188	——	——	——
C15:0	19.299	0.25±0.03^{c}	1.37±0.2^{b}	4.44±1.17^{a}
C16:1	20.136	——	4.57±0.82^{b}	45.14±7.80^{a}
C16:0	20.339	19.7±2.76^{c}	80.23±7.78^{b}	181.79±29.89^{a}
C17:1	21.273	0.22±0.02^{c}	1.19±0.11^{b}	5.81±1.3^{a}
C17:0	21.514	0.12±0.02^{c}	1.65±0.37^{b}	8.21±0.79^{a}
C18:3	22.288	1.2±0.24^{c}	14.64±3.26^{b}	53.59±11.36^{a}
C18 : 2n6t	22.511	——	——	——
C18 : 2n6c	22.607	24.11±2.76^{c}	181.54±13.02^{b}	948.81±117.84^{a}
C18 : 1	22.680	20.84±4.18^{c}	79.56±15.65^{b}	406.66±61.67^{a}
C18:0	22.975	5.63±0.55^{c}	19.66±3.52^{b}	84.64±12.99^{a}
C20 : 4n6	25.664	0.36±0.07^{c}	2.88±0.68^{b}	10.4±1.86^{a}
C20 : 5n3	25.819	0.15±0.02^{c}	1.37±0.27^{b}	4.75±0.89^{a}
C20:3n6/C20:3n3	26.07	0.11±0.02^{c}	0.36±0.11^{b}	1.94±0.48^{a}
C20:2	26.534	0.34±0.13^{c}	2.72±0.74^{b}	9.37±1.15^{a}
C20:1	26.665	0.14±0.01^{c}	1.51±0.22^{b}	6.19±0.53^{a}
C20:0	27.352	0.13±0.02^{c}	0.65±0.11^{b}	3.41±0.62^{a}
C21:0	30.708	——	0.42±0.05^{b}	1.73±0.71^{a}
C22 : 6n3	31.666	0.20±0.05^{c}	2.14±0.64^{b}	8.04±1.39^{a}
C22:2	33.721	0.13±0.01^{c}	0.52±0.06^{b}	1.93±0.42^{a}
C22:1n9	33.914	——	0.54±0.16^{b}	1.9±0.59^{a}
C22:0	35.22	——	0.4±0.04^{b}	1.7±0.25^{a}
C23:0	41.217	——	0.22±0.07^{b}	1.18±0.11^{a}
C24:1	44.554	——	0.39±0.09^{b}	1.59±0.39^{a}
C24:0	45.633	——	0.43±0.14^{b}	2.02±0.44^{a}
Total free fatty acids		83.12±7.31^{c}	556.94±54.84^{b}	2380.38±94.28^{a}

$^{a–c}$ Values with different superscripts differ significantly with respect to different digestive stage ($p < 0.05$).

IF1: vegetable oil-based formula; IF2: vegetable oil-based formula with MFGM; IF3: bovine milk/vegetable oil-based formula with cream; IF4 bovine milk/vegetable oil-based formula with cream and soybean phospholipid; IF5: whole fat milk power /vegetable oil-based formula.
